# A Rare Case of Myxoid Solitary Fibrous Tumor of the Upper Lip: The Key Role of STAT6 in Diagnosis

**DOI:** 10.7759/cureus.81510

**Published:** 2025-03-31

**Authors:** Benjamin Kahn, Francesca M Ceci, Faraz Yousefian, Jacqueline Nikakis, Matthew Elias, Marcus B Goodman

**Affiliations:** 1 Dermatology, Goodman Dermatology, Roswell, USA; 2 Dermatology, Peconic Bay Medical Center, Riverhead, USA; 3 Dermatology, Elias Dermatology, LLC, Fort Lauderdale, USA

**Keywords:** cutaneous oncology, lipoma, myxoid variation, neoplasm, solitary fibrous tumor

## Abstract

A solitary fibrous tumor (SFT) is an uncommon spindle cell mesenchymal neoplasm that most commonly arises in the pleura. SFTs have been shown to arise in various internal organs but are not typically discovered cutaneously. The diagnosis of SFT requires an integrated approach since SFT can mimic a variety of benign and malignant tumors, both clinically and histologically. To further complicate the histological diagnosis, there are different morphological variants of SFT. Therefore, immunohistochemical markers such as signal transducer and activator of transcription 6 (STAT6) can be utilized for SFT diagnosis due to their high specificity and sensitivity, as nuclear expression of STAT6 is a distinguishing feature of SFT and helps differentiate it from histological mimickers. A correct diagnosis is imperative for proper treatment and management. In this report, we present a 74-year-old healthy man with a growing painless mass on his upper lip for several years. Biopsy results confirmed the diagnosis of SFT with myxoid variation, and the patient underwent a successful excision of the lesion. Our case emphasizes the importance of a multidisciplinary approach and the utilization of immunohistochemical staining when diagnosing superficial tumors.

## Introduction

A solitary fibrous tumor (SFT) is a rare type of tumor that arises from connective tissue and is composed of spindle-shaped cells arranged in a collagen-rich background. Originally described in the pleura, SFTs can develop in various soft tissues, including the liver, kidney, central nervous system, thyroid, trachea, urogenital system, gastrointestinal tract, and, less commonly, the skin [1]. Cutaneous SFTs typically present as slow-growing, painless, well-circumscribed nodules, often on the extremities or head and neck. While most are benign, a small subset may exhibit aggressive behavior, with a potential for local recurrence or, rarely, malignant transformation.

Accurate diagnosis of cutaneous SFTs is critical due to their histological overlap with other spindle cell neoplasms, such as dermatofibrosarcoma protuberans and benign fibrous histiocytomas [2]. Moreover, although immunohistochemical markers such as CD34, CD99, and bcl-2 are commonly used to help diagnose SFT, these markers are nonspecific and are also expressed in other neoplasms that closely mimic SFT [3]. A key advancement in the diagnosis of SFTs has been the identification of a *NAB2-STAT6* gene fusion, leading to the nuclear overexpression of the signal transducer and activator of transcription 6 (STAT6) protein [1,2]. This discovery has made STAT6 immunohistochemistry a highly sensitive and specific diagnostic marker, distinguishing SFTs from other histologically similar tumors.

Although cutaneous SFT is uncommon, it is important that dermatologists and pathologists consider this diagnosis when evaluating primary spindle cell and/or myxoid neoplasms of the skin. We present a unique case of primary cutaneous SFT with a myxoid variation of the lip.

## Case presentation

**Figure 1:**
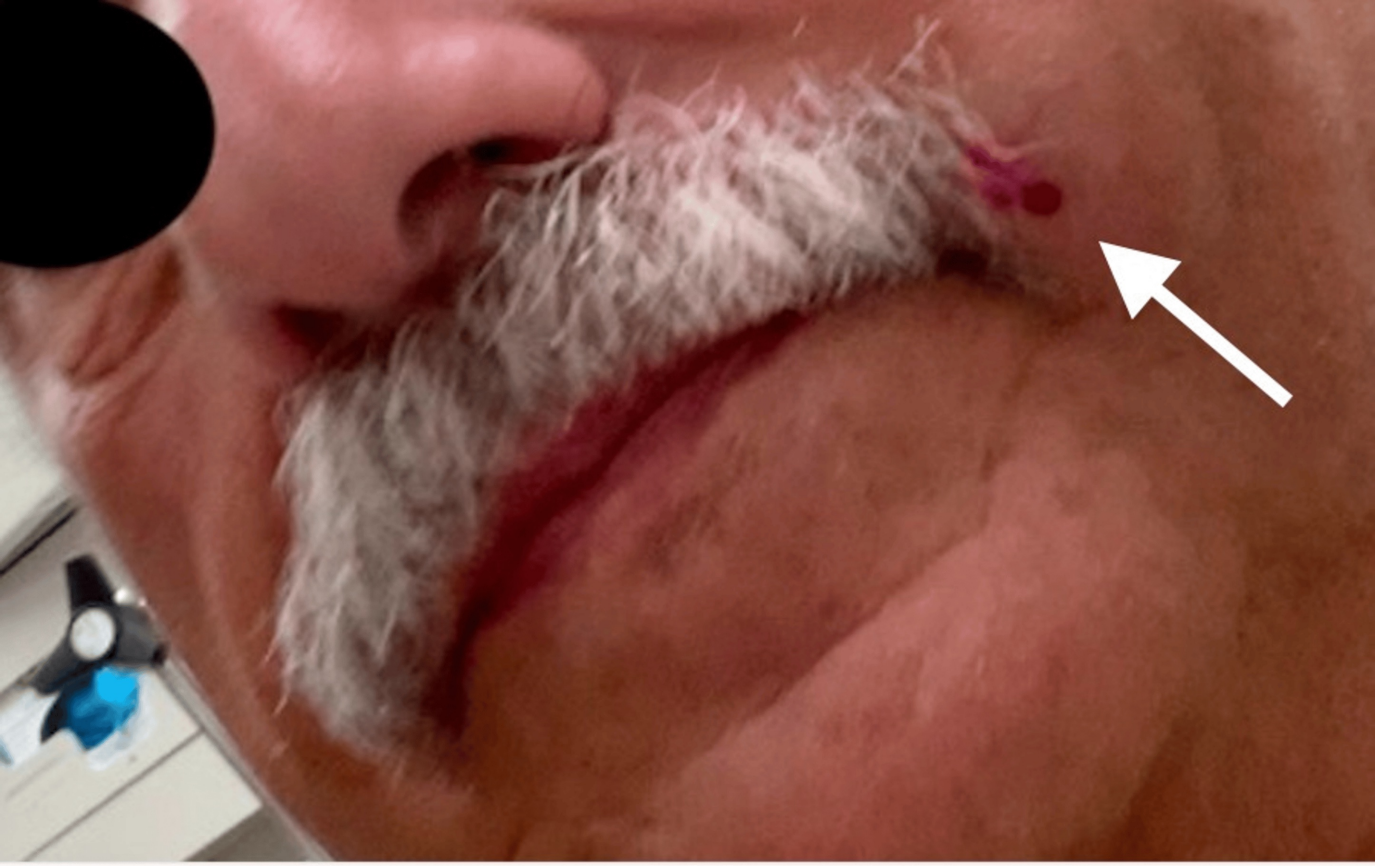
Nodule presentation A 5 mm, well-circumscribed, freely mobile, erythematous nodule on the upper lip.

A 74-year-old healthy man with no past medical history presented to the dermatology office complaining of a painless, slow-growing mass on his upper lip for "at least two years." Physical examination revealed a well-circumscribed, mobile, erythematous, 5 mm nodule on the left upper lip without epidermal changes (Figure 1). Examination also revealed no lymph node involvement.

**Figure 2:**
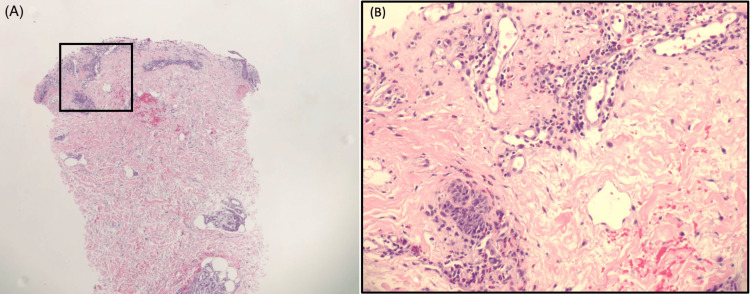
Hematoxylin and eosin staining A well-circumscribed tumor composed of short spindle cells with plump, elongated nuclei featuring tapered ends, fine chromatin, and eosinophilic cytoplasm with a myxoid variant. (A) Punch biopsy specimen showing skin with denuded epidermis and numerous eosinophils within the dermis (4×). (B) Higher magnification confirming the presence of numerous eosinophils (20×).

Following examination, a punch biopsy was performed. Histology revealed tumor cells arranged in fascicles that intersect one another in a patternless architecture with varying hypo- and hypercellular areas. The well-circumscribed tumor is composed of short spindle cells with plump elongated nuclei with tapered ends, fine chromatin, and eosinophilic cytoplasm with a myxoid variant. A prominent branching vascular tree with focal staghorn and hyalinized vessel walls was identified (Figure 2).

**Figure 3:**
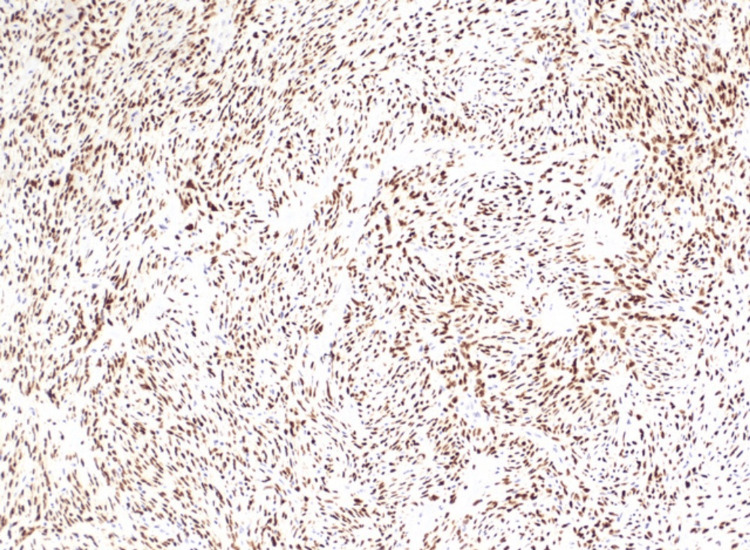
Immunohistochemical staining Immunohistochemical staining demonstrating STAT6 expression in neoplastic cells (10×). STAT6: signal transducer and activator of transcription 6

Immunohistochemical studies revealed diffuse vimentin and CD34 expression with focal CD63 expression. The neoplastic cells were negative for smooth muscle actin, SOX10, HMW keratin, keratin AE1.3, CD68, MOC-1, factor-13A, and desmin. No necrosis was present, and the mitotic count was 2/10 high-power fields. Based on histopathological and immunohistochemical results, differential diagnoses included hemangiopericytoma, giant cell angiofibroma, dermatofibrosarcoma protuberans, and solitary fibrous tumor. Furthermore, immunohistochemical evaluation revealed STAT6 expression of neoplastic cells (Figure 3).

These histological and immunohistochemical findings were consistent with a diagnosis of a myxoid variant of a solitary fibrous tumor.

At the one-week follow-up, the biopsy site was healing well. Education was provided regarding the diagnosis of SFT, and the patient was informed that excision is the current standard of care as recurrences may occur. The patient reported that he no longer feels the mass and would like to defer treatment at this time. The patient was also counseled on the importance of close long-term follow-up, as late occurrences can occur, and imagining such as CT scans may be used to monitor for recurrence [4].

## Discussion

Cutaneous SFTs are primarily seen in adults with a peak incidence in the fifth and sixth decades of life and have a female predominance [3,5]. These neoplasms have a predilection for the head and neck, with no identified risk factors for development. Clinically, cutaneous SFT typically presents as an asymptomatic, slow-growing tumor or nodule, often mistaken for a lipoma or cyst. Given its rarity, cutaneous SFTs can pose a diagnostic challenge, necessitating a careful histopathological and immunohistochemical workup to differentiate them from mimickers.

Histologically, SFTs are classically composed of spindle cells in a patternless arrangement with alternating hypo- and hypercellular areas, variable amounts of collagen, and "staghorn" or hemangiopericytic vasculature [2]. However, morphological variations, including giant-cell-rich, fibrous/fibromyxoid/angiofibromatous, myxoid/angiomyxoid, and lipomatous, can complicate diagnosis. A structured differential diagnosis is crucial in distinguishing SFT from these mimickers, as each entity carries different clinical implications and management strategies.

The varied histological appearance, coupled with its rarity within the skin, makes cutaneous SFT diagnosis particularly challenging. An integrated approach, including clinical, histological, immunohistochemical, and molecular findings, is required. Dermatologists and pathologists must be cognizant of cutaneous SFT, as it overlaps morphologically and immunophenotypically with other benign mesenchymal tumors and spindle cell sarcomas, such as spindle cell lipoma, benign and malignant nerve sheath tumors, and dermatofibrosarcoma protuberans [1]. STAT6 staining plays a pivotal role in differentiating SFTs from histological mimics, as nuclear STAT6 positivity is a key diagnostic feature identifying SFTs from tumors like dermatofibrosarcoma protuberans and myxoid liposarcoma. Our case reinforces the diagnostic utility of STAT6 staining in confirming the diagnosis of cutaneous SFT. Table 1 outlines the histopathological, immunohistochemical, and molecular features of SFT relative to its key differentials.

**Table 1:** Differential diagnosis for solitary fibrous tumors Differential diagnosis of solitary fibrous tumors and other cutaneous fibrohistiocytic and spindle cell tumors, with associated immunohistochemical markers [3,6-10]. COL1A-PDGFB: collagen type I alpha 1-platelet-derived growth factor beta, H3K27me3: histone H3 lysine 27 trimethylation, HMB-45: human melanoma black-45, HMGIC/HMGIY: high mobility group protein isoforms C and Y, INK4A: cyclin-dependent kinase inhibitor 2A, Ki-67: nuclear protein associated with proliferation, Leu-7: leucocyte antigen 7, MDM2: mouse double minute 2, NAB2: NGFI-A binding protein 2, NF1/NF2: neurofibromin 1/neurofibromin 2, NRAS: neuroblastoma RAS viral oncogene homolog, P16: cyclin-dependent kinase inhibitor 2A, PHF1: plant homeodomain finger protein 1, PLAG1: pleomorphic adenoma gene 1, PTCH: patched-1, PTEN: phosphatase and tensin homolog, RB1: retinoblastoma protein 1, S-100: soluble-100 protein, SOX10: SRY-box transcription factor 10, SUZ12/EED/PRC2: suppressor of zeste 12/embryonic ectoderm development/polycomb repressive complex 2, SYT-SSX1/2: synovial sarcoma translocation protein 1-2, SYT: synovial sarcoma translocation protein, TLE1: transducin-like enhancer of split 1, TP53: tumor protein p53, WT1: Wilms tumor protein 1, IHC: immunohistochemistry

Tumor	IHC	Key genetic alteration
Solitary fibrous tumor	CD34+, STAT6+, CD99+, nuclear β-catenin+, EMA+, BCL-2+	NAB2-STAT6 gene fusions
Spindle cell carcinoma	Cytokeratin+, vimentin+, CEA+, smooth muscle actin+, Ki-67+	RB1 deletion
Soft tissue angiofibroma	EMA−/+, CD34−/+	AHRR-NCOA2
Cellular angiofibroma	ER+ and PR+ (50%), CD34+/−, desmin−/+, ASMA −/+	RB1 deletion
Dermatofibrosarcoma protuberans	CD34+, ASAMA+/−, STAT6-	COLIA-PDGFB
Neurofibroma	S-100+, CD34+, +/- antibodies to EMA, CD57, and collagen IV, STAT6–/+	NF1/NF2 mutations
Malignant peripheral nerve sheath tumor	S100+	NF1, CDKN2A/CDKN2B, and PRC2 core components (EED or SUZ12) mutations
Myofibroma	ASMA+/−, CD34 −/+, desmin +	N/A
Myofibroblastoma	CD34+, Desmin + ASMA+/−	RB1 deletion
Leiomyoma	Vimentin+, smooth muscle actin+, desmin+	Dysregulations of HMGIC and HMGIY genes on chromosomes 6, 7, 12, and 14
Angioleiomyoma	Desmin+, SMA+, calponin+, h-caldesmon+	N/A
Hemangiopericytoma	CD34+, vimentin+, smooth muscle actin+/-, muscle-specific actin+/-, desmin-	NAB2-STAT6 gene fusions
Leiomyosarcoma	Desmin+, smooth muscle actin+	TP53, RB1 loss, ATRX, PTEN mutations
Spindle cell lipoma	CD34+, ASMA-, desmin-, S100-	RB1 deletion
Benign fibrous histiocytoma	Vimentin+, CD68+, -1-antitrypsin, -1-antichymotrypsin	FGFR-2 mutations
Dedifferentiated liposarcoma	MDM2+, CDK4+, p16+, CD34+/−, STAT6−/+	MDM2 and CDK4 amplification
Palisaded encapsulated neuroma	S-100+, vimentin+, GLUT1+, claudin 1+, EMA+/-	N/A
Neurilemmoma	S-100+, vimentin+, Leu-7 antigen+ and GFAP+, EMA+, fibronectin+, and collagen I and IlI	Schwann cell mutations
Cellular Schwannoma	S100+, SOX10+, STAT6-	NF2 mutations
Synovial sarcoma, monophasic	SYT +, TLE1+, EMA+, CK+, STAT6– (monophasic)/+	SYT-SSX1/2
Giant cell angiofibroma	CD34+, vimentin+, factor XIIIa+, S100 -	RB1, TP53, P16
Ossifying/fibromyxoid tumor	S-100+, desmin+, SMA+, STAT6 –/+	PHF1 gene rearrangement on chromosome 6p21
Deep fibrous histiocytoma	CD34 + (40%), ASMA f+/−, STAT6 – /+	N/A
Sarcomatoid mesothelioma	CK+, EMA+, D2–40+, Calretinin+, WT1+	BAP mutation
Pleomorphic adenoma/sarcoma	EMA+ (+/-) CEA+, cytokeratin+, vimentin+, S-100+, GFAP+, calponin+, PLAG 1+, STAT–/+	Gene 1 (PLAG1) rearrangement via chromosomal translocations involving 8q12
Kaposi sarcoma	CD31+ and CD34+	Viral DNA
Traumatic neuroma	S-100+, EMA+, CD57+, collagen IV	N/A
Rhabdomyosarcoma	Myoglobin+, desmin+, specific muscle-actin+	IGF2, ATR, PTCH, CDKN2A (P16 INK4A), CDKN2B, and TP53
Amelanotic melanoma	HMB-45+, S-100+, Melan-A+	BRAF, NRAS, CDKN2A, and NF1 mutations

While a combination of CD34, CD99, nuclear β-catenin, EMA, and BCL-2 has been widely used to help diagnose SFT, these markers are nonspecific [6]. The inclusion of molecular analysis, particularly STAT6 immunostaining and *NAB2-STAT6* gene fusion testing, has greatly improved diagnostic specificity. As seen in our case, the strong nuclear STAT6 positivity reinforced the diagnosis of cutaneous SFT, underscoring its diagnostic utility.

A recurrent chromosomal fusion affecting *NAB2* and *STAT6* genes was described in SFT [2]. This discovery led to the development of the highly sensitive and specific pathognomonic STAT6 immunohistochemical marker [3]. However, STAT6 positivity is not entirely exclusive to SFT, as it has been reported in a subset of soft tissue neoplasms (Table 1), including liposarcoma/myxoid liposarcoma, desmoid fibromatosis, deep fibrous histiocytoma, unclassifiable sarcoma, neurofibroma, undifferentiated pleomorphic sarcoma, low-grade fibromyxoid sarcoma, and ovarian fibroma [3,5].

While cutaneous SFTs are generally considered benign, locally aggressive behavior has been documented, with three reported cases of recurrence [2]. Although distant metastasis has not been observed in cutaneous SFT, histological transformation to a more aggressive phenotype has been reported in rare instances [11]. Thus, complete surgical excision with negative margins remains the gold standard, as deferring excision may increase the risk of local recurrence. However, ongoing surveillance is warranted to monitor for late recurrence.

Although focal myxoid change is a well-documented characteristic of SFT, extensive myxoid stroma is uncommon. Myxoid SFT was first described in 1999 as an SFT with myxoid change in at least 50% of the neoplasm [12]. While previous cases of primary cutaneous SFTs involving the lip have been reported, none have exhibited extensive myxoid change, making our case the first documented instance of primary cutaneous myxoid SFT of the lip. This highlights the importance of recognizing this rare histological variant.

Clinicians should maintain a high index of suspicion for cutaneous SFT when encountering a well-circumscribed, slow-growing nodule, particularly in the head and neck region. Awareness of these features aids in early diagnosis and appropriate management. In reviewing similar cases in the literature, we emphasize the need for clinicians to recognize the clinical signs of SFTs, particularly their well-circumscribed, slow-growing nature. Awareness of these features can aid in early diagnosis and appropriate management. By providing further insight into its histological and immunohistochemical presentation, along with clinical behavior and treatment outcomes, our case contributes to the broader understanding of this rare entity. Future studies exploring long-term outcomes and molecular markers may enhance prognostication and therapeutic strategies.

## Conclusions

SFTs are uncommon in the skin, making their recognition crucial for accurate diagnosis and management. While complete surgical excision is the standard of care, long-term follow-up is essential due to the potential for late recurrence or, in rare cases, malignant transformation. The recurrence risk for cutaneous SFTs remains low, but careful monitoring is advised, particularly for cases with atypical histological features.

Distinguishing SFT from other neoplasms can be challenging due to overlapping clinical features such as slow-growing, painless nodules and histological findings, including spindle cell morphology, myxoid stroma, and hemangiopericytoma-like vasculature. This case adds to the existing literature by highlighting the importance of STAT6 immunohistochemistry as a reliable diagnostic marker for cutaneous SFT. Given the histological overlap with other spindle cell neoplasms, integrating STAT6 into the diagnostic algorithm improves accuracy and reduces misclassification. Further research on long-term outcomes and the molecular underpinnings of cutaneous SFT could enhance our understanding of its clinical behavior and treatment implications.
